# Knockdown of cullin 4A inhibits growth and increases chemosensitivity in lung cancer cells

**DOI:** 10.1111/jcmm.12811

**Published:** 2016-03-10

**Authors:** Ming‐Szu Hung, I‐Chuan Chen, Liang You, David M. Jablons, Ya‐Chin Li, Jian‐Hua Mao, Zhidong Xu, Jr‐Hau Lung, Cheng‐Ta Yang, Shih‐Tung Liu

**Affiliations:** ^1^ Division of Thoracic OncologyDepartment of Pulmonary and Critical Care MedicineChang Gung Memorial HospitalChiayiTaiwan; ^2^ Department of MedicineCollege of MedicineChang Gung UniversityTaoyuanTaiwan; ^3^ Department of Respiratory CareChang Gung University of Science and TechnologyChiayiTaiwan; ^4^ Department of Emergency MedicineChang Gung Memorial HospitalChiayiTaiwan; ^5^ Department of NursingChang Gung University of Science and TechnologyChiayiTaiwan; ^6^ Thoracic Oncology LaboratoryDepartment of SurgeryComprehensive Cancer CenterUniversity of CaliforniaSan FranciscoCAUSA; ^7^ Life Sciences DivisionLawrence Berkeley National LaboratoryBerkeleyCAUSA; ^8^ Department of Medical Research and DevelopmentChang Gung Memorial HospitalChiayiTaiwan; ^9^ Department of Respiratory CareCollege of MedicineChang Gung UniversityTaoyuanTaiwan; ^10^ Department of Pulmonary and Critical Care MedicineChang Gung Memorial HospitalTaoyuanTaiwan; ^11^ Department of Microbiology and ImmunologyCollege of MedicineChang Gung UniversityTaoyuanTaiwan

**Keywords:** Cul4A, lung cancer, chemotherapy, p21

## Abstract

Cullin 4A (Cul4A) has been observed to be overexpressed in various cancers. In this study, the role of Cul4A in the growth and chemosensitivity in lung cancer cells were studied. We showed that Cul4A is overexpressed in lung cancer cells and tissues. Knockdown of the Cul4A expression by shRNA in lung cancer cells resulted in decreased cellular proliferation and growth in lung cancer cells. Increased sensitivity to gemcitabine, a chemotherapy drug, was also noted in those Cul4A knockdown lung cancer cells. Moreover, increased expression of p21, transforming growth factor (TGF)‐β inducible early gene‐1 (TIEG1) and TGF beta‐induced (TGFBI) was observed in lung cancer cells after Cul4A knockdown, which may be partially related to increased chemosensitivity to gemcitabine. G0/G1 cell cycle arrest was also noted after Cul4A knockdown. Notably, decreased tumour growth and increased chemosensitivity to gemcitabine were also noted after Cul4A knockdown in lung cancer xenograft nude mice models. In summary, our study showed that targeting Cul4A with RNAi or other techniques may provide a possible insight to the development of lung cancer therapy in the future.

## Introduction

Cullin 4A (Cul4A) is an 87‐kDa protein, which belongs to the family of evolutionally conserved cullin proteins, and has been shown to be related to ubiquitin proteosome pathway. Cul4A forms a part of the multifunctional ubiquitin‐protein ligase E3 complex by interacting with ring finger protein and damaged DNA‐binding protein 1. Through ubiquitin‐mediated proteolysis, Cul4A regulates many critical processes in cells. Cul4A ubiqutin ligase has been implicated to play important roles in: (*i*) cell cycle regulation 2; (*ii*) genome stability 3; (*iii*) nuclear excision repair 4; (*iv*) apoptosis 5 and (*v*) histone modification 6. Cul4A is also a critical gene for haematopoietic cell survival and development 7.

Previous studies have shown that Cul4A is amplified in malignant pleural mesothelioma 8, breast 9, lung cancer 10 and liver cancers 11. Overexpression of Cul4A has been reported in pituitary adenoma 12, prostate cancer 13 and osteosarcoma 14. Cul4A overexpression is associated with tumorigenesis of lung cancer 15 and hepatocellular carcinoma 16. Overexpression of Cul4A is related to breast cancer metastasis 17 and poor prognosis in ovarian cancer 18. Cul4A also has been implicated in the ubiquitination and proteolysis of tumour suppressors, such as p53 19, p21 20, p27 2, DLC1 21 and RASSF1A 22, and may contribute to tumorigenesis and cancer development through ubiquitination and then proteolysis of tumour suppressors. Recently, Cul4A has been reported to be associated with overexpression of Gli1, which is one of the critical transcription factors that mediate the Hh signalling pathway, in malignant pleural mesothelioma 23. In addition, Cul4A and ERK1/2 participate in multidrug resistance in breast cancer through regulation of multidrug resistance 1 gene (MDR1)/P‐gp expression 24. Knockdown of Cul4A is associated with increased chemosensitivity to cisplatin in lung cancer cells 15.

Because Cul4A is amplified and overexpressed in cancers and is related to cancer cell survival, growth, proliferation and chemosensitivity, we thus proposed that Cul4A may be a potential target of anticancer therapy. In this study, we attempted to elucidate the potential role of Cul4A in growth and tumorigenesis of lung cancer cells. Changes in drug susceptibility to common chemotherapy reagents after Cul4A knockdown by shRNA in lung cancer cells were also evaluated in our study.

## Materials and methods

### Cell lines and cell culture

H322, H460, A549, H838, H157, H1650, H1975 and H1703 lung cancer and WI‐38 normal lung cell lines were purchased from American Type Culture Collections (ATCC, Manassas, VA, USA). PC9 and HCC 827 lung cancer cells were generous gifts from Professor Pan‐Chyr Yang at National Taiwan University, Taipei, Taiwan. PC9, H1650, HCC827 and H1975 lung cancer cells have epidermal growth factor receptor(EGFR) mutations (delE746‐A750 for PC9, H1650 and HCC827; L858R and T790M for H1975). All cell lines were cultured in RPMI 1640 complete medium supplemented with 10% foetal bovine serum (FBS), penicillin (100 IU/ml) and streptomycin (100 μg/ml) and were cultured at 37°C and 5% CO_2_ in a humid incubator.

### Establishment of stable cells lines expression Cul4A shRNA and Cul4A‐myc

Cul4A shRNA, which was designed from a pre‐designed and pre‐validated Cul4A siRNA (Ambion, Austin, TX, USA) was cloned into the pSUPER.retro.puro vector (Oligoengine, Seattle, WA, USA) and retrovirus was produced as described previously 8. Cullin 4A shRNA targets the sequence: The pBABE‐puro (Addgene, Cambridge, MA, USA) retroviral vector was used to transduce the Cul4A gene 8 and myc‐tagged Cul4A (Cul4A‐myc) was overexpressed in H460, H157 and H322 lung cancer cells.

Retroviral infection was performed by adding filtered supernatant to lung cancer cell lines cultured on 10‐cm dishes with 50% confluent in the presence 8 ug/ml of polybrene (Sigma‐Aldrich, St. Louis, MO, USA). Six hours after infection, medium was changed with fresh medium and infected cells were allowed to recover for 48 hrs. Infected cells were selected by adding 10 μg/ml puromycin (Sigma‐Aldrich) to the culture medium for 48 hrs and then maintained in complete medium with 5 μg/ml puromycin.

### Tissues

To elucidate the clinical significance of Cul4A expression in lung cancer tissues, we studied the expression of Cul4A mRNA in 33 non‐small cell lung cancer (NSCLC) tumour and adjacent normal tissue samples after approval of the Institutional Review Board at Chang Gung Memorial Hospital. All samples are from early stage (stage I–III) NSCLC patients (Table S1) with informed consent signed and stored at −80°C in tissue bank of Chang Gung Memorial Hospital.

### Quantitative real‐time polymerase chain reaction

RNA was extracted from tumour and normal lung tissue samples and cDNA was then synthesized. Quantitative real‐time polymerase chain reaction was used to detect the expression of Cul4A mRNA with commercial Taqman probe (Applied Biosystems, Foster City, CA, USA). 18sRNA was used as internal control. The expression of Cul4A mRNA was normalized to the expression of a human normal lung bronchial epithelial cell line, HBEpiC (ScienCell Research Laboratories, San Diego, CA, USA) and the Bio‐Rad CFX96^™^ quantitative PCR system (Bio‐Rad Laboratories, Munich, Germany) was used.

### Cell proliferation assay

Cul4A shRNA was transfected by retrovirus into H157, H322 and H460 lung cancer cells. Stably transfected lung cancer cells (1 × 10^4^) were plated in 6‐well culture plates and incubated in complete medium. Cells were counted each day with an automated cell counter (Beckman Coulter, Brea, CA, USA).

### Colony formation assay

For anchorage‐dependent colony formation assay, stably transfected (5 × 10^2^) lung cancer cells were plated in 10‐cm culture dishes and incubated in complete medium for 14 days. The colonies were then stained with 0.1% crystal violet, and colonies with more than 50 cells were counted.

For anchorage‐independent growth assay, soft agar colony formation assay was performed to detect malignant transformation of cells. Cells were cultured in DMEM plus 15% FBS in 0.35% (w/v) low‐melting‐temperature agar between layers of 0.7% low‐melting‐temperature agar. After 4 weeks, colonies were stained with 3‐(4, 5‐dimethylthiazol‐2‐yl)‐2,5‐ diphenyltetrazolium bromide (Sigma Chemical Co., Saint Louis, MO, USA ), and colonies containing more than 100 cells were scored. Colonies were photographed and counted after staining.

### Chemosensitivity assay

H157, H460 and H322 lung cancer stable cells (1 × 10^4^) were cultured in 6‐well plates for 48 hrs and then treated with indicated concentrations of gemcitabine. Seventy‐two hours after being treated with indicated concentrations of gemcitabine, cisplatin or pemetrexed, cell numbers were determined using a cell counter. The IC_50_ value was determined using GraphPad Prism^®^ log (inhibitor) *versus* response (variable slope) software (version 5; La Jolla, CA, USA).

### Apoptosis assay

Apoptosis assay (Annexin‐V FITC) was performed in lung cancer cell lines after treated with 5 μM gemcitabine for 72 hours. The occurrence of apoptosis was determined by ApoTarget^™^ annexin V‐FITC kit (BioSource International, Inc., Camarillo, CA, USA) according to manufacturer's manual with a flow cytometer.

### Western blot analysis

Whole protein was extracted by M‐PER Mammalian Protein Extraction Reagent added with Phosphatase Inhibitor Cocktail Set II (Calbiochem, San Diego, CA, USA) and Complete Protease Inhibitor Cocktails (Roche, Lewes, UK). Proteins were separated on 7.5% SDS‐PAGE and transferred to Immobilon‐P membranes (Millipore, Billerica, MA, USA). The following primary antibodies were used: Cul4A (Abcam, Cambridge, MA, USA), cleaved PARP antibody (Cell Signaling, Danvers, MA, USA), p21 (Santa Cruz Biotechnology, Santa Cruz, CA, USA), TGF‐β inducible early gene‐1 (TIEG1; Abcam), transforming growth factor, beta‐induced (TGFBI; Abcam) and β‐actin (Sigma‐Aldrich). After antigen–antibody complexes were bound to secondary antibodies, an enhanced chemiluminescence blotting analysis system (GE Healthcare Life Sciences, Piscataway, NJ, USA) was used to detect antigen–antibody complexes.

### Cell cycle analysis

Cell cycle analysis was performed with propidium iodide stain. Briefly, Cells were harvested with trypsin and then washed twice in PBS. 1 × 10^6^ cells were fixed in ice‐cold 70% ethanol overnight. Cells were then washed twice in PBS/1% BSA, treated with 500 μg/ml RNase for 30 min. at 37°C, then stained with 50 μg/ml propidium iodide overnight at 4°C. Cells were analysed on a flow cytometer.

### Transfection of siRNA and cell viability assay

For cell viability assay, 1 × 10^3^ lung cancer cells were cultured in a 96‐well plate for 48 hrs. Lung cancer cells were transfected with 50 nM of control or pre‐designed and pre‐validated p21 siRNA 25 (sc‐29427; Santa Cruz Biotechnology) for 48 hrs using Lipofectamine^™^ RNAiMAX Transfection Reagent (Invitrogen, Carlsbad, CA, USA), according to the manufacturer's protocol, and then treated with 10 nM of gemcitabine for 72 hrs. Survival cells were determined by CellTiter‐Glo luminescent cell viability assay (Promega, Madison, WI, USA) using EnSpire^®^ Multimode Plate Reader (PerkinElmer, Waltham, MA, USA).

For Cul4A siRNA transfection, a pre‐designed and validated ON‐TARGET plus Cul4A siRNA (Dharmacon, Lafayette, CO, USA) which targets a different sequence of human Cul4A mRNA (GCACAGAUCCUUCCGUUUA) from Cul4A shRNA as previously (GGUUUAUCCACGGUAAAGA) 8 used. Cells were plated in 60‐mm dishes in antibiotic‐free media. Transfection was performed with cells at 60% confluence with a final concentration of 50 nM for each siRNA as described previously. At 72 hrs after transfection, cells were harvested and analysed for protein expression.

### Transfection of Cul4A‐myc in H1975 lung cancer cells

For transient transfection of Cul4A‐myc in H1975 lung cancer cells, cells were plated in 60‐mm dishes in antibiotic‐free media. Transfection was performed with cells at 60% confluence with pcDNA3‐myc3‐CUL4A (Addgene) or empty pcDNA3 (Invitrogen) vectors using Lipofectamine 2000 transfection reagent (Invitrogen). At 72 hrs after transfection, cells were harvested and analysed for protein expression, anchorage‐dependent colony formation and gemcitabine inhibition assays as described previously.

### Xenograft mice model

After approval of the Institutional Animal Care and Use Committee at Chang Gung Memorial Hospital, 1 × 10^6^ cells in PBS were mixed with ice‐cold matrigel (BD Biosciences, San Jose, CA, USA) in a total volume of 200 μl and were injected subcutaneously into the flank area of each 6–8‐week‐old female BALB/C nude mouse. Tumours were measured once to twice a week using callipers, and the tumour volume was calculated as Volume = *W* ^2^ × *L*/2, where *L* was the longest diameter and *W* was its perpendicular width.

For gemcitabine treatment, therapy was initiated when the volume of tumours reached 300–500 mm^3^. Gemcitabine (120 mg/kg) in normal saline with a total volume of 100 μl was injected intraperitoneally twice a week for 2 weeks. Relative tumour volume was calculated according to the following formula: *RTV* = *TVn*/*TV*0, where *TVn* is the tumour volume at day *n* and *TV*0 is the tumour volume at day 0.

### Statistical analysis

All statistical analyses were performed with GraphPad Prism^®^ (version 5). The data shown represent mean values ± S.D. Student's *t*‐test was used to compare results between control and experimental groups unless otherwise specified. Two‐sided *P*‐values 0.05 were considered significant.

## Results

### Cul4A is overexpressed in lung cancer cell lines and tissues

**Figure 1:**
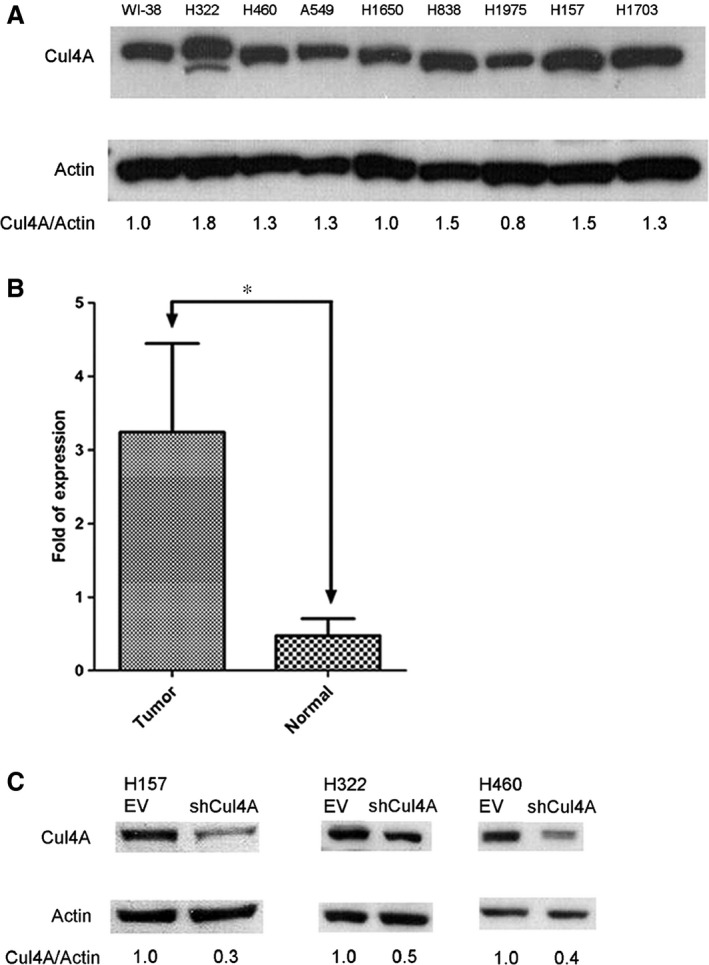
(**A**) Expression of Cul4A in normal lung and lung cancer cell lines. Cul4A in normal lung (WI‐38) and lung cancer (H322, H460, A549, H1650, H838, H1975, H157 and H1703) cell lines. Internal control: β‐Actin. Density of Cul4A bands was quantified and normalized to β‐Actin using WI‐38 cell line as a normal control. (**B**) Expression of Cul4A mRNA in NSCLC tumour and paired normal lung tissue samples. Bars represent the average of fold of expression ± standard deviation (*n* = 32). ‘*’ denotes *P* < 0.0001, *t*‐test. (**C**) Knockdown of Cul4A expression by shRNA. Density of Cul4A bands was quantified and normalized to β‐Actin using empty vector‐transfected cell lines as normal controls. EV: empty vector; shRNA: Cul4A shRNA.

We first investigated the expression of Cul4A in lung cancer cell lines and found that overexpression of Cul4A was noted in six lung cancer cell lines (H322, H460, A549, H838, H157 and H1703) out of eight cell lines compared to the expression of Cul4A in a normal lung cell line (WI‐38) (Fig. 1A). Since two cell lines without overexpression of Cul4A are EGFR mutant cell lines (H1650 and H1975), we further checked the expression of Cul4A in additional two EGFR mutant cell lines (PC9 and HCC827). Lower expression of Cul4A was observed in PC9 and HCC827 lung cancer cell lines compared to normal lung cell line (WI‐38) (Fig. S1A).

RNA was extracted from tumour and normal lung tissue samples and cDNA was then synthesized. A significantly increased expression of Cul4A mRNA was noted in NSCLC tumour compared to paired normal lung tissue samples (Fig. 1B). Recently, overexpression of Cul4A was reported in NSCLC tissues 10 and the same findings were also observed in our study.

### Knockdown of Cul4A inhibits growth in lung cancer cells

**Figure 2:**
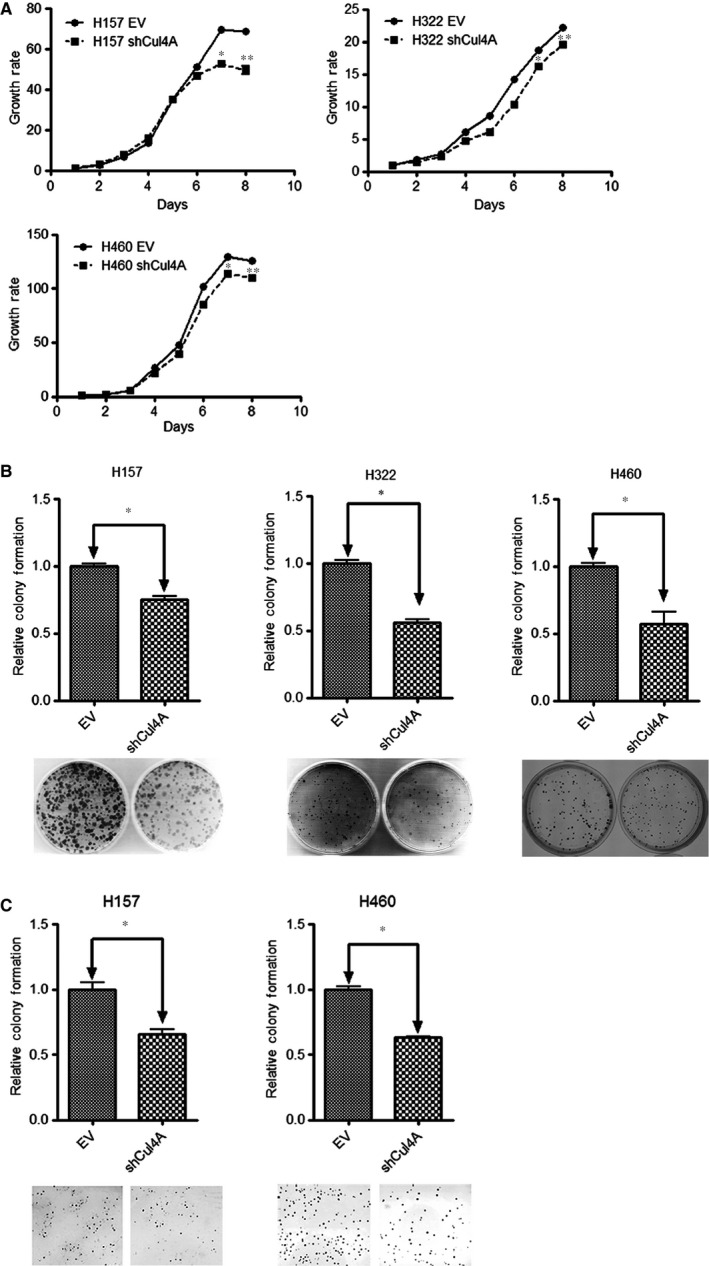
(**A**) Cell proliferation assay in Cul4AshRNA knockdown lung cancer cell lines. Growth rate of the cell is represented by normalization of the cell count on each day to the cell count on day one in each group of cell line studied and shown as bar ± standard deviation in triplet experiments. ‘*’ and ‘**’ denotes *P* < 0.05 on day 7 and day 8, *t*‐test. EV: empty vector; shRNA: Cul4A shRNA. (**B**) Anchorage‐dependent colony formation assay in Cul4AshRNA knockdown H460, H157 and H322 lung cancer cell lines. Relative colony formation is represented by normalization of the colony number to the empty vector transfected cell line and shown as bar ± standard deviation in triplet experiments. EV: empty vector; Cul4A: Cul4A shRNA; ‘*’ denotes *P* < 0.05, *t*‐test. (**C**) Anchorage independent soft agar colony formation assay in Cul4AshRNA knockdown H460 and H157 lung cancer cell lines. Relative colony formation is represented by normalization of the colony number to the empty vector transfected cell line and shown as bar ± standard deviation in triplet experiments. EV: empty vector; Cul4A: Cul4A shRNA; ‘*’ denotes *P* < 0.05, *t*‐test.

To establish stable Cul4A knockdown cells, retrovirus expressing Cul4A shRNA was established as previously described 8. Cul4A knockdown was performed by retrovirus‐transfected shRNA in H157, H322 and H460 lung cancer cells (Fig. 1C). Decreased expression (50–70%) of Cul4A after shRNA knockdown was noted in the lung cancer cells studied. In H157, H322 and H460 lung cancer cells, knockdown of Cul4A inhibited proliferation (Fig. 2A) of lung cancer cells. Moreover, knockdown of Cul4A also inhibited anchorage‐dependent and anchorage‐independent colony formation assays (Fig. 2B and C) of cancer cells. Our results showed that knockdown of Cul4A is associated with growth inhibition in lung cancer cells.

### Knockdown of Cul4A increases chemosensitiviy in lung cancer cells

**Figure 3:**
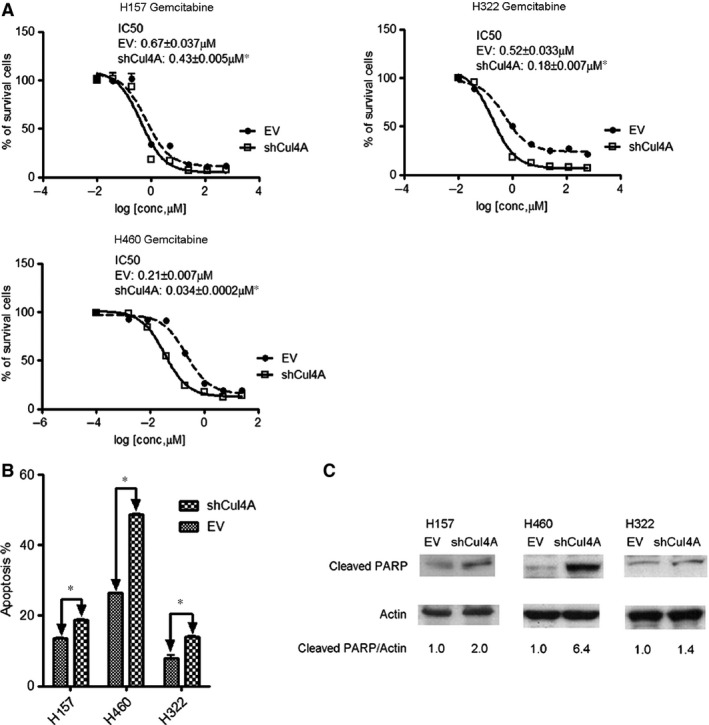
(**A**) IC_50_ values for gemcitabine in Cul4A shRNA transfected H157, H460, and H322 lung cancer cell lines. Data points represent the average of IC_50_ value± standard deviation of gemcitabine in triplet experiments. EV: empty vector; Cul4A: Cul4A shRNA; ‘*’ denotes *P* < 0.05, *t*‐test. (**B**) Apoptosis assay (Annexin‐V FITC) in lung cancer cell lines after treated with 5 μM gemcitabine for 72 hrs. Percentage of apoptotic cells were shown as bar ± standard deviation in triplet experiments. EV: empty vector; Cul4A: Cul4A shRNA; ‘*’ denotes *P* < 0.05, *t*‐test. (**C**) Western blot analysis for cleaved PARP. Lung cancer cell lines were treated with 5 μM gemcitabine for 24 hrs and were harvested. Total cell lysate was used for Western blot analysis with cleaved PARP antibody (Cell Signaling). Density of cleaved PARP bands were quantified and normalized to β‐Actin using empty vector transfected cell lines as normal controls. EV: empty vector; Cul4A: Cul4A shRNA.

With the purpose to evaluate the association of Cul4A and drug sensitivity to chemotherapy drugs in lung cancer cells, we treated Cul4A shRNA transfected stable H157, H322 and H460 lung cancer cells with gemcitabine, which is a chemotherapy agent. Gemcitabine is a pyrimidine nucleoside antimetabolite agent which is active in several human malignancies, including NSCLC 26. When compared to IC_50_ values of gemcitabine in empty vector‐transfected control cell lines, significantly lower IC_50_ values (Fig. 3A) were observed in Cul4A shRNA knockdown cell lines. Increased apoptotic cells were noted in Cul4A knockdown stable cell lines after treatment with gemcitabine (Fig. 3B), which was further confirmed by increased expression of cleaved PARP in Cul4A knockdown stable cell lines treated with gemcitabine (Fig. 3C).

Increased chemosensitivity to cisplatin was also observed in Cul4A knockdown H157, H322 and H460 lung cancer cells (Fig. S3A). Increased chemosensitivity to pemetrexed was observed in Cul4A knockdown H460 lung cancer cells (Fig. S3B). Less chemosensitivity to pemetrexed was observed in Cul4A knockdown H157 lung cancer cells. IC_50_ values in Cul4A knockdown H157 lung cancer cells were not determined, but significantly growth inhibition to pemetrexed was observed (Fig. S3B).

### Knockdown of Cul4A up‐regulates expression of p21, TIEG1 and TGFBI in lung cancer cells

**Figure 4:**
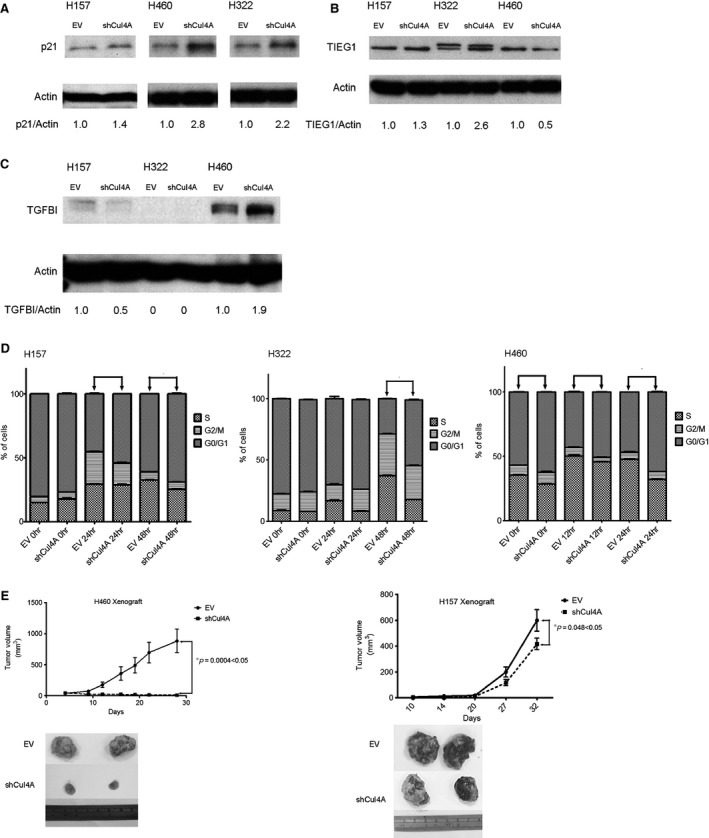
Cul4A shRNA was transfected by retrovirus into H157, H322 and H460 lung cancer cell lines. Stable cells transfected with Cul4A shRNA were harvested and total cell lysate was used for Western blot analysis. (**A**) Western blot analysis for p21 in lung cancer cell lines. Density of p21 bands was quantified and normalized to β‐Actin using empty vector‐transfected cell lines as normal controls. EV: empty vector; Cul4A: Cul4A shRNA. (**B**) Western blot analysis for TIEG1 in lung cancer cell lines. Density of TIEG1 bands were quantified and normalized to β‐Actin using empty vector transfected cell lines as normal controls. EV: empty vector; Cul4A: Cul4A shRNA. (**C**) Western blot analysis for TGFBI in lung cancer cell lines. Density of TGFBI bands were quantified and normalized to actin using empty vector‐transfected cell lines as normal controls. EV: empty vector; Cul4A: Cul4A shRNA. (**D**) Cell cycle analysis for lung cancer cell lines. ‘*’ denotes *P* < 0.05, *t*‐test. (**E**) Knockdown of Cul4A decreased the growth of tumours in H460 (*n* = 8 in each group) and H157 (*n* = 6 in each group) xenograft nude mice models. 1 × 10^6^ cells in PBS were mixed with ice‐cold matrigel (BD Biosciences) in a total volume of 200 μl and were injected subcutaneously into the flank area of each 6–8‐week‐old female BALB/C nude mouse. Tumours were measured once to twice a week using callipers, and the tumour volume was calculated as Volume = *W* ^2^ × *L*/2, where *L* was the longest diameter and *W* was its perpendicular width. Data points represent the average of tumour volume± standard deviation. EV: empty vector; Cul4A: Cul4A shRNA; ‘*’ denotes *P* < 0.05, *t*‐test.

We further investigated the possible mechanisms of the oncogenic role of Cul4A through which Cul4A regulates the protein level of cell cycle‐dependent kinase inhibitors and we observed that knockdown of Cul4A increased expression levels of the cell cycle‐dependent kinase inhibitors and tumour suppressor p21 (Fig. 4A). Through literature review, we found that increased expression of TIEG1 27 and TGFBI 28 proteins were related to increased sensitivity to gemcitabine. Thus, we studied the expression of TIEG1 and TGFBI in Cul4A shRNA transfected stable lung cancer cells. We found that knockdown of Cul4A also resulted in increased expression levels of TIEG1 in H157 and H322 lung cancer cells (Fig. 4B). Additionally, knockdown of Cul4A increased expression levels of TGFBI in H460 lung cancer cells (Fig. 4C). Our results were verified using another Cul4A siRNA targeting a different sequence of Cul4A mRNA. Consistent with stable lung cancer cells, increased expression of p21 were observed in H157, H322 and H460 lung cancer cells (Fig. S2). Increased TIEG1 was observed in H157 and H322 (Fig. S2A and B) and increased TGFBI (Fig. S2C) in H460 in lung cancer cells.

Up‐regulation of p21 was also observed in lung cancer cells after Cul4A knockdown in our study. As a result, we also evaluated changes of cell cycle after Cul4A knockdown in lung cancer cells and G0/G1 cell cycle arrest was also observed in the lung cancer cells studied (Fig. 4D).

### Knockdown of Cul4A inhibits tumour growth in murine lung cancer xenograft models

With the purpose to observe the growth of lung tumours after Cul4A knockdown, lung cancer xenograft models using H157 and H460 stable lung cancer cells were established. Significantly decreased tumour growth after Cul4A knockdown was observed in both H460 and H157 lung cancer cells (Fig. 4).

### Overexpression of Cul4A down‐regulates expression of p21, TIEG1 and TGFBI in lung cancer cells

**Figure 5:**
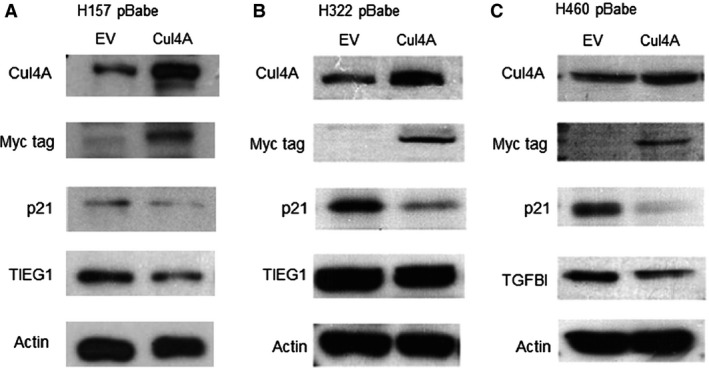
The pBABE‐puro (Addgene) retroviral vector was used to transduce the Cul4A gene and myc‐tagged Cul4A (Cul4A‐myc) was overexpressed in H157, H322 and H460 lung cancer cells. (**A** and **B**) Western blot analysis for Cul4A, Myc‐tag, p21, TIEG1 and actin in H157 and H322 lung cancer cells. (**C**) Western blot analysis for Cul4A, Myc‐tag, p21, TGFBI and actin in H460 lung cancer cells. EV: empty vector; Cul4A: Cul4A‐myc.

To further validate the above findings after Cul4A knockdown, overexpression of Cul4A study was performed. Overexpression of Cul4A in lung cancer cells were performed in retroviral transfection of Myc‐tagged Cul4A in H460, H157 and H322 lung cancer cells using pBABE‐puro vector (Fig. 5). Transient overexpression of Myc‐tagged Cul4A was also performed in H1975 lung cancer cells (Fig. S1B). On the contrary, p21 was down‐regulated in all cells studied after overexpression of Cul4A (Fig. 5, Fig. S1B). Down‐regulation of TGFBI was observed in H460 and H1975 lung cancer cells (Fig. 5C, Fig. S1B), and of TIEG1 in H157 and H322 (Fig. 5A and B) lung cancer cells. Different expressions of TIEG1 and TGFBI in lung cancer cell lines after Cul4A knockdown or expression may indicate different pathways for gemcitabine chemosensitivity in different lung cancer cells.

### Overexpression of Cul4A decreases chemosensitiviy in lung cancer cells

**Figure 6:**
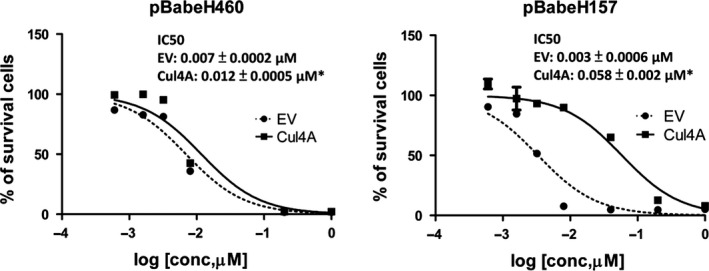
IC_50_ values for gemcitabine in Cul4A overexpressed (**A**) H460 and (**B**) H157 lung cancer cells. Cells (1 × 10^4^) were cultured in 6‐well plates for 48 hrs and then treated with indicated concentrations of gemcitabine for 96 hrs. Data points represent the average of IC_50_ value± standard deviation of gemcitabine in triplet experiments. EV: empty vector; Cul4A: Cul4A‐myc; ‘*’ denotes *P* < 0.05, *t*‐test.

We further studied chemosensitivity to gemcitabine after Cul4A overexpression in H460 and H157 lung cancer cells and observed that significantly increased IC_50_ was observed in these two cell lines compared to empty virus transfected controls (Fig. 6A and B). In H1975 lung cancer cells, overexpression of Cul4A was also associated with increased anchorage‐dependent colony formation (Fig. S1C) and decreased chemosensitivity to gemcitabine (Fig. S1D).

### Increased chemosensitivity after knockdown of Cul4A is partially regulated by p21

**Figure 7:**
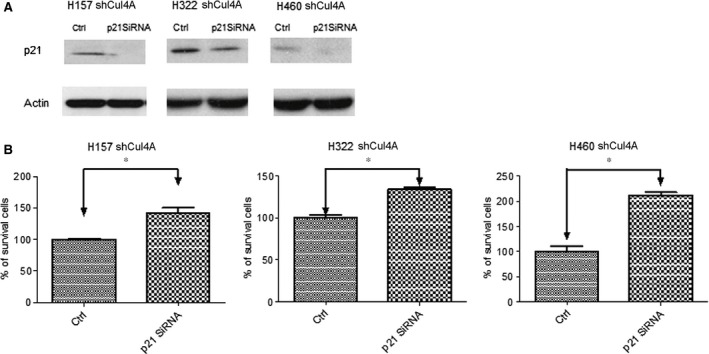
(**A**) Western blot analysis of p21 in H157, H322 and H460 lung cancer cells lung cancer cells after transfection of p21 siRNA. All lung cancer cells were transfected with pSuper Cul4A for down‐regulation of Cul4A. Actin was used as the internal control. (**B**) Inhibition of gemcitabine to H157, H322 and H460 lung cancer cells studied. The percentage of survival cells was normalized to groups without gemcitabine treatment and shown as bar ± standard deviation in triplet experiments. * denotes *P* < 0.05. Ctrl: control siRNA.

To further study the mechanism of increased chemosensitivity after knockdown of Cul4A, p21 siRNA was transfected to the Cul4A knockdown lung cancer cells (Fig. 7A). Notably, knockdown of p21 by siRNA was observed to decrease chemosensitivity in Cul4A knockdown H157, H157 and H460 lung cancer cells (Fig. 7B). Increased chemosensitivity after Cul4A knockdown may be partially related to the up‐regulation of p21 in lung cancer cells.

### Knockdown of Cul4A increases chemosensitiviy in murine lung cancer xenograft models

**Figure 8:**
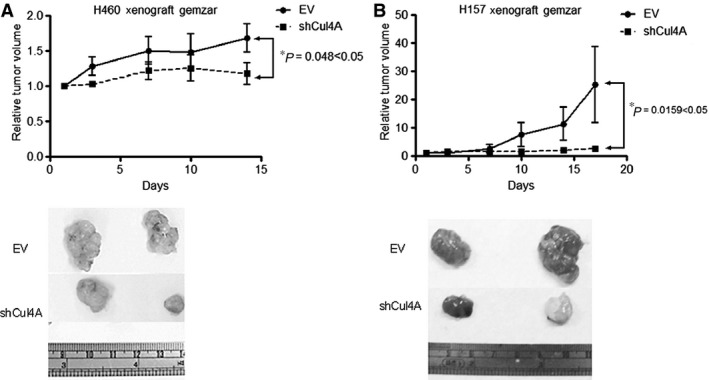
Knockdown of Cul4A increased chemosensitivity to gemcitabine in (**A**) H460 and (**B**) H157 xenograft nude mice models. Data points represent the average of relative tumour volume ± standard deviation (*n* = 4 in each group). ‘*’ denotes *P* < 0.05, two‐way anova test.

Because knockdown of Cul4A increased chemosensitivity to gemcitabine in H460 and H157 lung cancer cell lines, we studied the effects of Cul4A knockdown on chemosensitivity to gemcitabine in H460 and H157 xenograft models. Significant growth inhibition by gemcitabine was observed in Cul4A knockdown H460 (Fig. 8A) and H157 tumours (Fig. 8B).

## Discussion

In our previous study, we observed that Cul4A knockdown is associated with G0/G1 cell cycle arrest through up‐regulation of p21 in mesothelioma cells 8. In transgenic mice study, conditional overexpression of Cul4A is associated with decreased p21 expression in lung adenocarcinoma 15. Through *in vitro* and *in vivo* studies, we observed that Cul4A is an important regulator of proliferation, cell cycle progression and tumour growth in lung cancer in this study. In addition, we also observed that increased chemosensitivity to gemcitabine was observed after knockdown of Cul4A in lung cancer cells and vice versa.

The mechanism of increased sensitivity to gemcitabine after knockdown of Cul4A in lung cancer cell lines remains unknown. Our preliminary results showed that it could be related to increased cyclin‐dependent kinase inhibitor p21/WAF1, TIEG1 and TGFBI proteins. The p21 plays a role as a cell cycle‐dependent kinase inhibitor in cellular process. Deficiency of p21 is associated with abrogation of cells to undergo G1 arrest following DNA damage in p21 knockout mice 29 and colon cancer cells 30. Overexpression of p21 in hamster BHK21 cells causes cell cycle arrest in the G1 phase, and reduces cell growth and DNA synthesis 31. The p21 also plays a role as a tumour suppressor protein, and up‐regulation of p21 has been reported to increase chemosensitivity in cancer cells 32.

TIEG1 is a Krupel‐like zinc finger transcription factor regulating cellular growth and differentiation. TIEG1 is an early response gene to the stimulation of TGF‐β1 33. Overexpression of TIEG1 is reported to promote apoptosis induced by a proteasome inhibitor, Valcade, in leukaemic cells 34. Overexpression of TIEG1 has been reported to increase apoptosis in both TGF‐β‐sensitive and resistant cancer cells and concurrently enhanced sensitivity to gemcitabine 27. TGFBI is known as keratoepithelin or βIg‐h3, which is a 68‐kD protein, and it contains four conserved fasciclin‐1 domains and a carboxyl‐terminal Arg‐Gly‐Asp (RGD) integrin‐binding sequence. TGFBI mediates integrin binding to extracellular matrix proteins such as collagen, laminin and fibronectin 35. Loss of TGFBI expression has been reported in several cancers including lung carcinoma 36, and it has been suggested to act as a tumour suppressor gene 37. In addition, overexpression of TGFBI in lung cancer cells has been reported to be associated with increased sensitivity to gemcitabine 28. In our study, up‐regulation of TIEG1 was noted in H157 and H322 lung cancer cell lines, and up‐regulation of TGFBI was noted in H460 lung cancer cell line. In H460 lung cancer cell line, higher expression of TGFBI was noted compared to the expression of TGFBI in H157 and H322 lung cancer cell lines, which may account for increased sensitivity to gemcitabine after knockdown of Cul4A in H460 lung cancer cell line. On the contrary, lower expression of TGFBI was noted in H157 and H322 lung cancer cell lines, and up‐regulation of TIEG1 may account for increased sensitivity to gemcitabine after knockdown of Cul4A in these two cancer cell lines. TIEG1 has been reported as a transactivator of TGFBI in renal cell carcinoma 38; however, the interaction between TIEG1 and TGFBI in lung cancer cells remains unclear. In our study, different lung cancer cells showed different expressions of TIEG1 and TGFBI after Cul4A knockdown or expression, which may indicate different pathways for gemcitabine chemosensitivity in different lung cancer cells. Further studies to elucidate the association of Cul4A with TGFBI and TIEG1 in lung cancer cells are ongoing in our laboratory.

Increased chemosensitivity to cisplatin after Cul4A knockdown was also observed in our study, which is compatible to our previous study 15. Platinum‐based chemotherapy remains the most commonly used regimen in the treatment of lung cancer 39 to date. Our results may provide another evidence that targeting Cul4A may add to increase chemosensitivity in platinum‐based chemotherapy in lung cancer patients. In the contrary, increased chemosensitivity to pemetrexed was only observed in H460 lung cancer cells in our study. Our results indicate that Cul4A may regulate chemosensitivity through various mechanisms in different lung cancer cells and further studies are warranted.

Although our findings are similar to a previous study 10, which emphasized on the overexpression of Cul4A with enhanced growth of lung cancer cells and increased sensitivity to erlotinib, which is a target therapy drug for lung cancer. In our work, we emphasized the effects of Cul4A knockdown on lung cancer growth and increased chemosensitivity to gemcitabine, cisplatin and pemetrexed, which are a commonly used chemotherapy drugs, in lung cancer *in vitro* and *in vivo*. We further extended our study to lung bronchioalveolar cell carcinoma (H322), large cell carcinoma (H460) and squamous cell carcinoma (H157) cells. Overexpression of Cul4A was observed in both studies. Together with a previous study, the importance of Cul4A in the tumorigenesis, and most importantly, the value of Cul4A as a therapeutic target was further addressed, at least in Asian patients.

In summary, our study showed that Cul4A plays important roles in cell growth and survival in lung cancer cells and knockdown of Cul4A is associated with increased chemosensitivity to gemcitabine. As a result, targeting Cul4A with small molecules, RNAi, or other techniques may provide a possible insight to the development of lung cancer therapy as well as other cancers. However, further study is still needed to elucidate the underlying mechanisms of Cul4A knockdown and the associated increased chemosensitivity.

## Conflicts of interest

The authors have no declared conflicts of interest.

## Supporting information


**Figure S1** (**A**) Western blot analysis of Cul4A and actin in normal lung (WI‐38) and lung cancer cells (PC9, and HCC827). (**B**) Western blot analysis for Cul4A, Myc‐tag, p21, TGFBI, TIEG1 and actin in H1975 lung cancer cells overexpressed with Cul4A‐myc. (**C**) Anchorage‐dependent colony formation assay in Cul4A‐myc overexpressed H1975 lung cancer cells. Relative colony formation is represented by normalization of the colony number to the empty vector transfected cell line and shown as bar ± standard deviation in triplet experiments. (**D**) Inhibition of gemcitabine to H1975 lung cancer cells studied. The percentage of survival cells was normalized to groups without gemcitabine treatment and shown as bar ± standard deviation in triplet experiments. EV: empty vector; Cul4A: Cul4A‐myc; ‘*’ denotes *P* < 0.05, *t*‐test.

**Figure S2** Transient transfection of Cul4A siRNA in H157, H322 and H460 lung cancer cells. (**A** and **B**) Western blot analysis for Cul4A, Myc‐tag, p21, TIEG1 and actin in H157 and H322 lung cancer cells. (**C**) Western blot analysis for Cul4A, Myc‐tag, p21, TGFBI and actin in H460 lung cancer cells. EV: empty vector; Cul4A: Cul4A‐myc. Ctrl: control siRNA.

**Figure S3** IC_50_ values for (**A**) cisplatin and (**B**) Alimta in Cul4A shRNA transfected H157, H460, and H322 lung cancer cells. Data points represent the average of IC_50_ value± standard deviation in triplet experiments. EV: empty vector; Cul4A: Cul4A shRNA; ‘*’ denotes *P* < 0.05, *t*‐test. ND: not determined.

**Table S1** Clinical characteristics of 33 NSCLC patients.
