# Supplementary figures and images for: Knockdown of cullin 4A inhibits growth and increases chemosensitivity in lung cancer cells
Source: J Cell Mol Med. 2016 Mar 10;20(7):1295–306. doi: 10.1111/jcmm.12811 (PMC4929302; doi:10.1111/jcmm.12811)

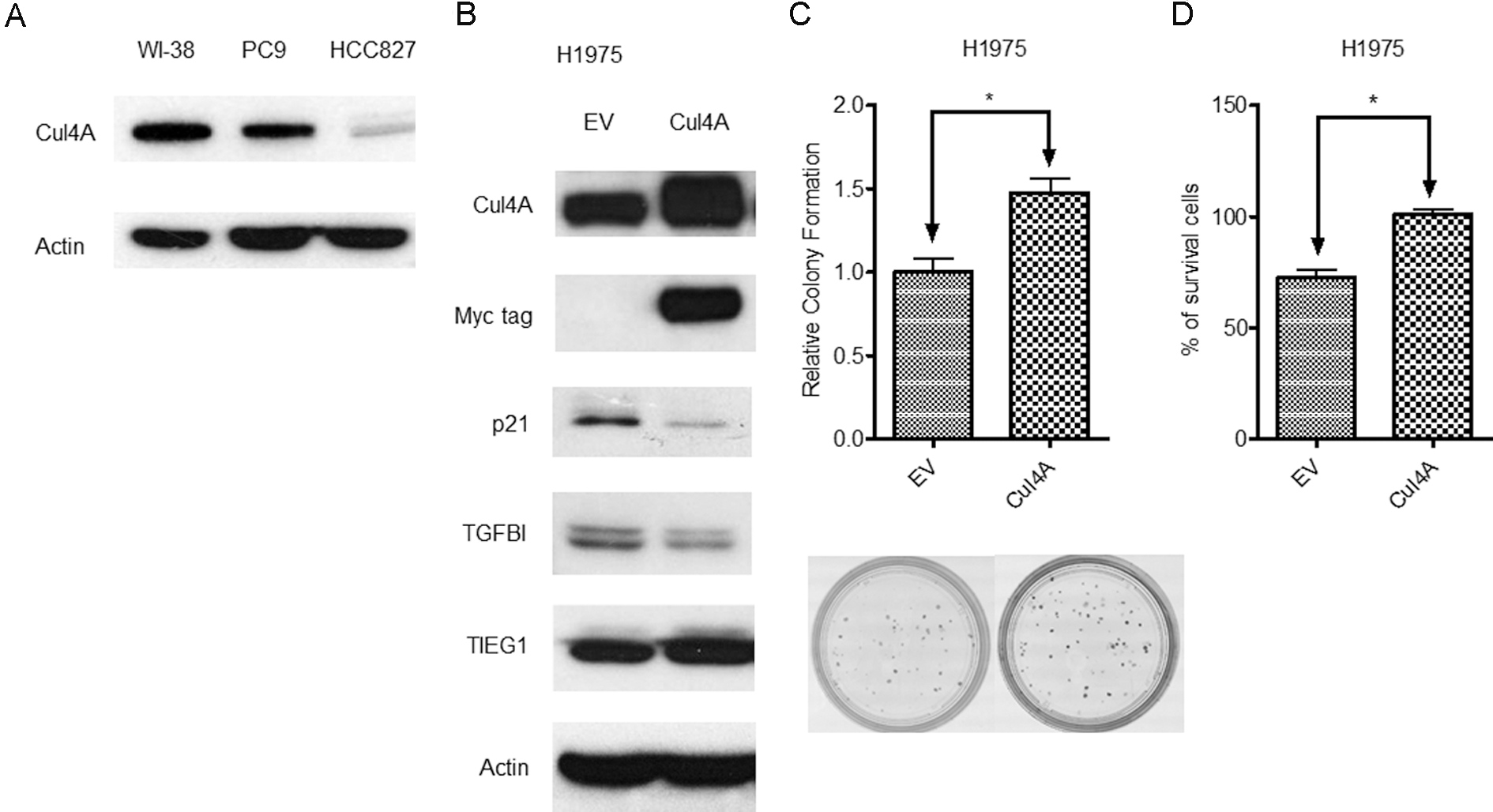

Supplement: Supplementary file 1 — Figure S1 (A) Western blot analysis of Cul4A and actin in normal lung (WI‐38) and lung cancer cells (PC9, and HCC827). (B) Western blot analysis for Cul4A, Myc‐tag, p21, TGFBI, TIEG1 and actin in H1975 lung cancer cells overexpressed with Cul4A‐myc. (C) Anchorage‐dependent colony formation assay in Cul4A‐myc overexpressed H1975 lung cancer cells. Relative colony formation is represented by normalization of the colony number to the empty vector transfected cell line and shown as bar ± standard deviation in triplet experiments. (D) Inhibition of gemcitabine to H1975 lung cancer cells studied. The percentage of survival cells was normalized to groups without gemcitabine treatment and shown as bar ± standard deviation in triplet experiments. EV: empty vector; Cul4A: Cul4A‐myc; ‘*’ denotes P < 0.05, t‐test. [file JCMM-20-1295-s001.tiff]

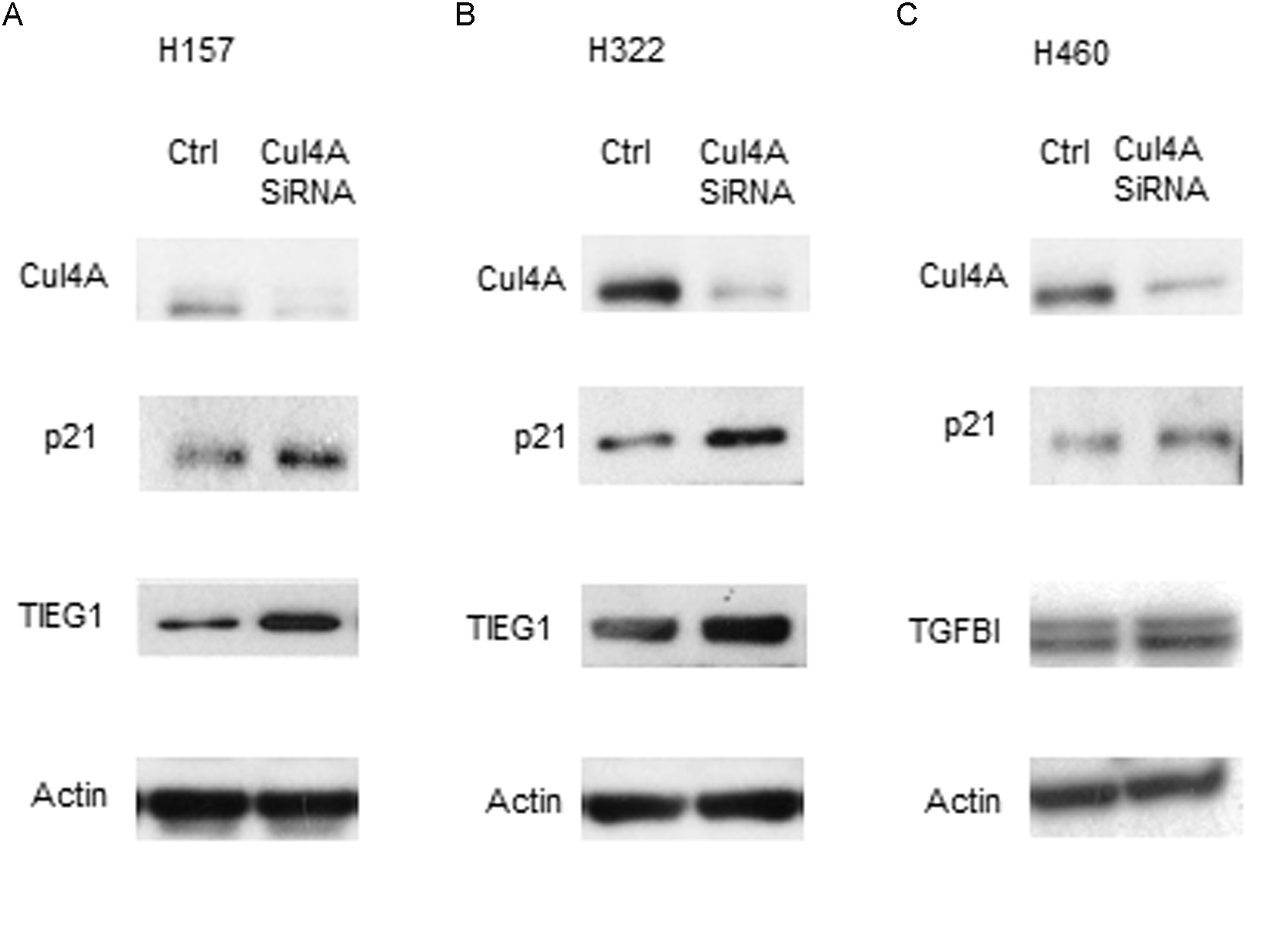

Supplement: Supplementary file 2 — Figure S2 Transient transfection of Cul4A siRNA in H157, H322 and H460 lung cancer cells. (A and B) Western blot analysis for Cul4A, Myc‐tag, p21, TIEG1 and actin in H157 and H322 lung cancer cells. (C) Western blot analysis for Cul4A, Myc‐tag, p21, TGFBI and actin in H460 lung cancer cells. EV: empty vector; Cul4A: Cul4A‐myc. Ctrl: control siRNA. [file JCMM-20-1295-s002.tiff]

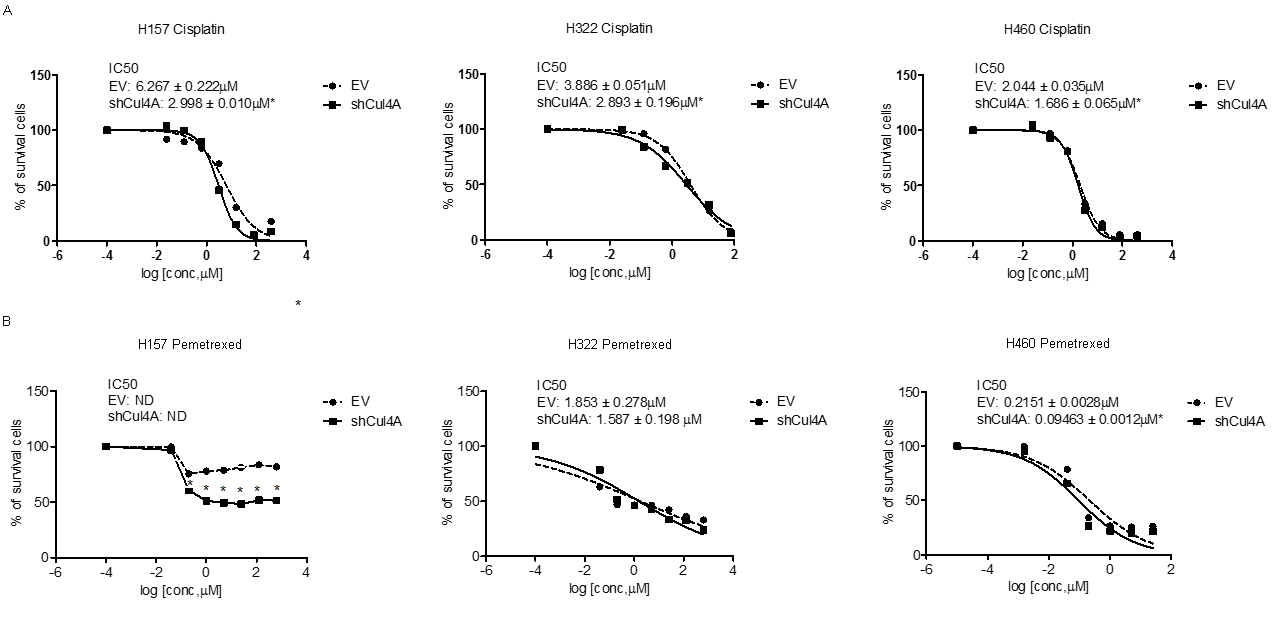

Supplement: Supplementary file 3 — Figure S3 IC50 values for (A) cisplatin and (B) Alimta in Cul4A shRNA transfected H157, H460, and H322 lung cancer cells. Data points represent the average of IC50 value± standard deviation in triplet experiments. EV: empty vector; Cul4A: Cul4A shRNA; ‘*’ denotes P < 0.05, t‐test. ND: not determined. [file JCMM-20-1295-s003.tiff]
