# Supplementary material for: Knockdown of cullin 4A inhibits growth and increases chemosensitivity in lung cancer cells
Source: J Cell Mol Med. 2016 Mar 10;20(7):1295–306. doi: 10.1111/jcmm.12811 (PMC4929302; doi:10.1111/jcmm.12811)
Supplement: Supplementary file 4 — Table S1 Clinical characteristics of 33 NSCLC patients. [file JCMM-20-1295-s004.doc]

Table S1. Clinical characteristics of 33 NSCLC patients

| **Clinical characteristics** |  |
| --- | --- |
| **Medain age (year)** | 62.7 |
| **Gender** |  |
| Male | 22 (66.7%) |
| Female | 11 (33.3%) |
| **Stage** |  |
| I | 20 (66.6%) |
| II | 6 (18.2%) |
| III | 6 (18.2%) |
| IV | 1 (3.0%) |
| **Pathology** |  |
| Adenocarcinoma | 21 (63.6%) |
| Squamous carcinoma | 6 (18.2%) |
| Adenosquamous carcinoma | 2 (6.1%) |
| Large cell carcinoma | 3 (9.1%) |
| Sarcomatoid | 1 (3.0%) |
